# A modified SDS-based DNA extraction method from raw soybean

**DOI:** 10.1042/BSR20182271

**Published:** 2019-02-05

**Authors:** Yimiao Xia, Fusheng Chen, Yan Du, Chen Liu, Guanhao Bu, Ying Xin, Boye Liu

**Affiliations:** College of Food Science and Technology, Henan University of Technology, Zhengzhou 450001, Henan, China

**Keywords:** DNA extraction, GMO detection, Soybean, SDS method

## Abstract

Soybean is the most important genetically modified (GM) oilseed worldwide. Regulations relating to the approval of biotech soybean varieties and product labeling demand accurate and reliable detection techniques to screen for GM soya. High-quality extracted DNA is essential for DNA-based monitoring methods. Thus, four widely used protocols (SDS, CTAB, DP305, and DNeasy Plant Mini Kit) were compared in the present study to explore the most efficient DNA extraction method for raw soya matrix. The SDS-based method showed the highest applicability. Then crucial factors influencing DNA yield and purity, such as SDS lysis buffer component concentrations and organic compounds used to isolate DNA, were further investigated to improve the DNA obtained from raw soybean seeds, which accounts for the innovation of this work. As a result, lysis buffer (2% SDS (w/v), 150 mM NaCl, 50 mM Tris/HCl, 50 mM EDTA, pH 8.0) and organic reagents including chloroform/isoamyl alcohol (24:1, v/v) (C: I), isopropanol, and ethanol corresponding to the extraction and first and second precipitation procedures, respectively, were used in the optimized SDS method. The optimized method was verified by extracting approximately 2020–2444 ng DNA/mg soybean with A_260/280_ ratios of 1.862–1.954 from five biotech and non-biotech soybean varieties. Only 0.5 mg of soya was required to obtain enough DNA for PCR amplification using the optimized SDS-based method. These results indicate that the screening protocol in the present study achieves the highest suitability and efficiency for DNA isolation from raw soya seed flour.

## Introduction

Soybean (*Glycine max*) plays a dominant role in human nutrition and as a protein source for animal feed. In 2017, soybean oilseed production reached 351.32 million tons, accounting for approximately 60% of global oilseed yield [1]. Moreover, 91.4 million hectares of biotech soybean in 2017 occupied 50% of the global biotech crop area and 77% of the global soybean cultivation area [2]. Soybean is clearly the most important biotech oilseed worldwide.

Consumption of genetically modified (GM) food has raised consumer concerns widely, across Europe, Asia, and so on. As a result, a series of regulations and laws have been published to limit the quantity allowed in food and to standardize labeling rules [3,4]. To guarantee the implementation of the rules, an accurate, efficient, and reliable analysis method is needed to allow the detection of GM material in food mixtures.

Detection methods for GM material monitor the introduced DNA and its corresponding proteins. Native protein structure is always denatured by the pressure, shearing, heating, and acid and alkali treatments in food processing, easily resulting in false negatives for protein-based detection methods. In contrast, DNA-based methods have high sensitivity and specificity, which allows detection in raw or highly processed foods [5–7]. However, soya matrix contains protein, oil, polysaccharide, polyphenol, and other contaminants, which inhibit PCR assays [6]. To facilitate DNA amplification, it is critical to isolate high purity DNA from this complicated food mixture with a suitable and efficient DNA extraction protocol.

SDS, CTAB, and multiple commercial kits have been used to purify DNA from soya material [7–9]. So far, the SDS-based method has been reported to enable the highest DNA yield from soya matrix [10,11]. However, we found the concentration of components in SDS lysis buffers varied widely, detailed in Table 1. Additionally, specific DNA yields were not shown clearly in most of these reports. Thus, the influence of the lysis buffer composition on DNA yield was impossible to figure out, resulting in variable usage during SDS-based DNA extraction. Moreover, the organic reagents used to remove contaminants and precipitate DNA also vary between DNA isolation protocols, and are rarely compared [12,13].

**Table 1:** The concentrations of components in SDS lysis buffers

Samples	SDS lysis buffer components				pH	References
	SDS (m/v, %)	NaCl (mM)	Tris/HCl (mM)	EDTA (mM)		
Soybean sauce, milk	0.5	250	200	25	8.0	[3]
Broken pieces of soybean	0.5	288	200	25	/	[8]
Soybean powder	0.5	0	10	5	/	[10]
Soybean coat, cotyledon, embryo	0.5	500	100	50	/	[11]
Soybean	8	300	60	30	/	[14]
Soybean	3	0	50	50	8.0	[15]
Soybean, meal, powder	1.4	500	100	50	/	[16]

Therefore, the objectives of this work were to study the specific effects of SDS lysis buffer composition and organic compounds on the quality and quantity of extracted DNA. To our knowledge, no research has yet focussed on optimizing the SDS extraction method step-by-step to improve DNA quality from raw soya matrix.

## Materials and methods

### Soybean seed sources

Five kinds of soybean seeds including non-biotech and biotech varieties were utilized in the present study. The non-biotech soybean seed materials, Zhoudou22, Zheng196, and Zhonghuang13, were purchased from Henan Academy of Agricultural Sciences. Two kinds of GM soybean seeds were obtained from Henan Sunshine Oils and Fats Group importing GM materials from America annually. Immunochromatography test strip assay had been performed revealing 5-enolpyruvyl-shikimate-3-phosphate synthase (EPSPS) positive for both the GM soybean seeds. Considering the GM soybean samples were geographically different, we named them Roundup Ready Soybean (RRS) 1 (RRS1) and Roundup Ready Soybean2 (RRS2) respectively.

### Genomic DNA extraction protocols

All soya seeds were ground and homogenized in liquid nitrogen with a mixer mill, followed by filtrating through 80-mesh sieve. All ground samples were stored at −20°C prior to DNA extraction.

#### SDS method 1

One hundred milligram of Zhoudou22 was weighted and transferred to a 2-ml sterile centrifuge tube. One milliliter of SDS extraction buffer (20 g SDS/l, 150 mM NaCl, 100 mM Tris/HCl, 25 mM EDTA, pH 8.0) preheated at 65°C was added and mixed followed by adding 10 μl Proteinase K (10 mg/ml). Then, the reaction tube was incubated at 65°C for 1 h, with stirring every 10 min. After centrifuging the tube for 10 min at 12000×***g***, the supernatant was extracted twice with phenol/chloroform/isoamyl alcohol (P: C: I, 25: 24: 1, v/v/v) (first extraction) and chloroform/isoamyl alcohol (C: I, 24: 1, v/v) (second extraction), respectively. Then the upper aqueous phase was added with 0.1 volume potassium acetate solution (3 M, pH 5.5) and double volume of ethanol solution (95%, v/v, −20°C) (first precipitation), followed by gentle inversion and vortex for 10 min at 15000×***g*** to pellet DNA. After washing the pellet with ethanol solution (70%, v/v, −20°C) twice and air drying for 5 min, the dried pellet was dissolved with 400 μl Tris/EDTA buffer (10 mM Tris, 1 mM EDTA). Ten milligram of RNase was added in the mixture and an incubation at 37°C for 30 min was performed to eliminate the remaining RNA. Another extraction with C:I (third extraction) was carried out to remove protein from DNA solution. Recovering the upper layer to a new sterile tube containing 2.5 vol of ethanol (second precipitation) would help precipitate DNA readily. After spinning tube at 15000×***g*** for 10 min and washing DNA pellet twice, the dried DNA was redissolved in 200 μl sterile, deionized water.

#### CTAB method

The operation in this method was similar to that described in SDS method 1 except the lysis buffer and organic regents used to precipitate DNA. This method was based on CTAB extraction buffer (20 g CTAB/l, 1.4 M NaCl, 100 mM Tris/HCl, 20 mM EDTA, pH 8.0) and 0.6 vol of isopropanol was added to the upper aqueous phase to pellet DNA twice instead of ethanol solution used in SDS method 1.

#### DP305 and DNeasy plant mini kit method

Concerning two commercial kits, DP305 (TIANGEN, Beijing, China) and DNeasy Plant Mini kit (Qiagen, Hilden, Germany), DNA was extracted from Zhoudou22 according to the manufacturer’s instructions.

#### SDS method 2

The DNA extraction protocol was described in SDS method 1 except for the lysis buffer components. Concentrations of each component in SDS extraction buffer are detailed in Table 2.

**Table 2:** Components composition of lysis buffer and organic reagents used in SDS-based DNA extraction

Components composition of SDS lysis buffer (pH 8.0)				
Number	SDS (w/v)	NaCl (mM)	Tris/HCl (mM)	EDTA (mM)
1	0.5%	250	100	25
2	2%	250	100	25
3	5%	250	100	25
4	2%	0	100	25
5	2%	150	100	25
6	2%	250	100	25
7	2%	500	100	25
8	2%	250	10	25
9	2%	250	50	25
10	2%	250	100	25
11	2%	250	200	25
12	2%	250	100	5
13	2%	250	100	25
14	2%	250	100	50
15	2%	250	100	100

Organic reagents used to extract and precipitate DNA				
Procedure	First extraction	Third extraction	First precipitation	Second precipitation
A	P: C: I	C: I	Ethanol	Ethanol
B	C: I	C: I	Ethanol	Ethanol
C	P: C: I	/	Ethanol	Eethanol
D	P: C: I	C: I	KAc + 95% Ethanol	Ethanol
E	P: C: I	C: I	Isopropanol	Ethanol
F	C: I	C: I	Ethanol	Isopropanol
G	C: I	C: I	Isopropanol	Isopropanol
H	C: I	C: I	Isopropanol	Ethanol

#### SDS method 3

The DNA extraction protocol was described in SDS method 1 except for the organic solvent varieties. The use of organic solvents was shown in Table 2. The addition volume of isopropanol was 0.6 vol supernatant.

#### SDS method 4

It was the optimized DNA extraction method. Its protocol was shown in SDS method 1 except several modifications: SDS lysis buffer (2% SDS (w/v), 150 mM NaCl, 50 mM Tris/HCl, 50 mM EDTA, pH 8.0), first extraction reagent (C: I), third extraction reagent (C: I), first precipitation reagent (isopropanol), second precipitation reagent (ethanol).

## DNA quantitation and purity

DNA yield and purity were determined by using Nanodrop 2000 (Thermo Scientific, Waltham, U.S.A.), a UV spectrophotometry method, at 260 nm absorption value and absorbance ratios at 260/280 nm respectively.

### Primers design and qualitative PCR

Routine PCR was carried out to screen *cp4epsps* gene and intrinsic *lectin* gene of soybean. Primer pairs, Lectin-F/R 1, and Epsps-F/R 1, were used by previous studies [5,17]. Other primers were designed using software Primer Premier 5 and Oligo 7 based on genomic sequences of the *lectin* gene (GenBank accession: **K00821**) and *Glycine max* transgenic *cp4epsps* gene for EPSPS class 2 precursor (GenBank accession: **AB209952**). All primers were synthesized by Sangon Biotech (Shanghai, China). Sequences of primers and amplification conditions were detailed in Table 3. The conventional PCRs were performed in 25 μl total reaction volume containing 1× Premix Taq™ (TaKaRa Bio Co., Beijing, China), 0.12 μM of each primer, and 80 ng template DNA. Amplifications were carried out in TECHNE TC-412 Thermal cycler (Staffordshire, U.K.) using 5 min denaturation at 95°C initially, then 35 cycles of 30 s at 94°C, 30 s at a specific annealing temperature (Table 3), certain extension time (Table 3) at 72°C, followed by a final extension of 10 min at 72°C.

**Table 3:** Primers and amplification conditions used in qualitative PCR

Target	Primer	Sequence (5′–3′)	Amplicon size (bp)	Annealing temperature (°C)	Extension time (s)	References
*lectin*	Lectin-F1	GCC CTC TAC TCC ACC CCC ATC C	118	58	20	[17]
	Lectin-R1	GCC CAT CTG CAA GCC TTT TTG TG				
	Lectin-F2	TGC CGA AGC AAC CAA ACA TGA TCC T	438	56	40	This work
	Lectin-R2	TGA TGG ATC TGA TAG AAT TGA CGT T				
	Lectin-F3	GGC AAA CTC AGC GGA AAC TGT	772	55	60	This work
	Lectin-R3	TTA GAT GGC CTC ATG CAA CAC				
	Lectin-F4	ACC CTT GTT AGT CAA ACC ACA	1067	55	75	This work
	Lectin-R4	AAC CCT ATC CTC ACC CAC TCG				
*cp4epsps*	Epsps-F1	ATC CCA CTA TCC TTC GCA AGA	169	53	20	[5]
	Epsps-R1	TGG GGT TTA TGG AAA TTG GAA				
	Epsps-F2	CCT TCA TGT TCG GCG GTC TCG	498	62	40	This work
	Epsps-R2	GCG TCA TGA TCG GCT CGA TG				
	Epsps-F3	TTC ATC GGC GAC GCC TCG CTC ACA	700	64	60	This work
	Epsps-R3	CGC GGA GTT CTT CCA GAC CGT TCA T				
	Epsps-F4	CGC CCG CAA ATC CTC TGG CCT TTC	1099	64	75	This work
	Epsps-R4	CGT CTC GCC CTC ATC GCA ATC CAC				

### Agarose gel electrophoresis

The quality of DNA was studied by electrophoresis in a 1% agarose gel (Sangon Biotech, Shanghai, China) containing 4S Green Plus Nucleic Acid Stain (Sangon Biotech, Shanghai, China) in 1× TAE buffer. The amplification products were separated by 2% agarose gel. The agarose gel image was visualized and recorded using a UV Bio-Rad Gel Doc 2000 image detector (Bio-Rad, Hercules, U.S.A.) installing analysis software Quantity one.

### Suitability test of the optimized DNA extraction method

SDS method 4 (the optimized method) was conducted to extract gDNA from Zhoudou22, Zheng196, Zhonghuang13, RRS1 and RRS2 to confirm the suitability of the optimized method.

### Sensitivity of the optimized DNA extraction method

SDS method 4 (the optimized method) was applied to a range of RRS1 (100, 50, 10, 5, 1, and 0.5 mg) to extract DNA to evaluate the minimum RRS1 quantity used for DNA extraction and conventional PCR examination.

### Data analysis

Each sample was tested in triplicate. One-way ANOVA analysis was conducted using SPSS software version 21.0 (IBM Analytics, Armonk, U.S.A.) to test the significance of differences amongst DNA yields.

## Results and discussion

### Qualitative analysis of DNA extracted with four methods

**Figure 1:**
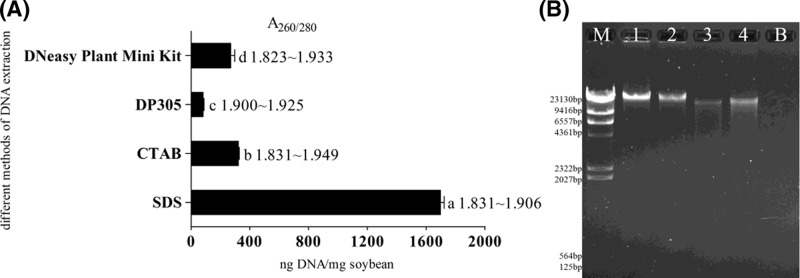
Comparison of four different DNA extraction methods A_260/280_ ratios of DNA extracted with different methods; different lowercase letters indicate significant differences amongst DNA yields in (**A**); M, 1, 2, 3, 4, and B correspond to λ DNA HindIII Marker (TIANGEN, Beijing, China), SDS-based method, CTAB method, DP305 method, DNeasy Plant Mini Kit, and negative control PCR in (**B**).

We compared four universal methods (SDS, CTAB, DP305 kit, and DNeasy Plant Mini Kit) to determine the most suitable procedure to isolate DNA from soya material. There were significant differences amongst the different DNA extraction methods (Figure 1A). The SDS-based method gave the highest DNA yield from soybean seeds, while the other methods had considerably lower DNA yields. Similar results are also shown in Figure 1B. Although DNA yields differed significantly, DNA purity was high (2.0 > A_260/280_ ratio > 1.8) regardless of the extraction method. Extraction of DNA from soybean seeds with the SDS-based method produced the highest purity and concentration.

Similarly, Wang et al. [16] reported that the SDS-based method was most suitable for extracting DNA from less processed soybean-derived products (seed, meal, powder) after comparing the DNA yields and purity achieved from CTAB, SDS, and TaKaRa kit extraction protocols. In addition, the yields from raw soybean and derivatives using the SDS-based method fluctuated between 975 and 1025 ng/mg [16], proximate to our results (Figure 1A), whereas the reports from Kamiya and Kiguchi [10] and Demekea et al. [8] contained lower DNA yields (100–300 ng/mg and 100 ng/mg) from soybean seeds using the SDS-based extraction method. Differences amongst the DNA yields were probably caused by the distinct SDS-based lysis buffer compositions and organic reagents used to isolate or precipitate DNA. The effect of varying specific factors in the SDS-based method is detailed in ‘Optimization of SDS lysis buffer composition’ and ‘Optimization of organic reagents used in DNA extraction’ sections.

A few other reports have demonstrated that the CTAB method gives higher DNA yields for complex foodstuffs and difficult samples [7,18,19], therefore, the use of raw soya matrices in our study accounts for the lower concentrations obtained by CTAB extractions. DNeasy Plant Mini Kit and CTAB DNA extraction methods were also performed on raw soybean seeds by Stefanova et al. [9] and Wang et al. [16], with a lower genomic DNA yield (50–150 ng/mg) than this study (270–322 ng/mg) (Figure 1). Moreover, several commercial DNA extraction kits not studied in this report were also used to evaluate the suitability and efficiency of different protocols on less processed soya materials. The DNA yields from the NucleoSpin Food kit, GeneSpin, Wizard, and Fast ID Genomic DNA Extraction Kit were approximately 150, 50–160, 100, and 100 ng/mg respectively [7–9]. Accordingly, there seems to be a tendency for SDS-based methods to produce higher concentrations of DNA from raw soybean seeds when compared with commercial extraction kits.

### Optimization of SDS lysis buffer composition

**Figure 2:**
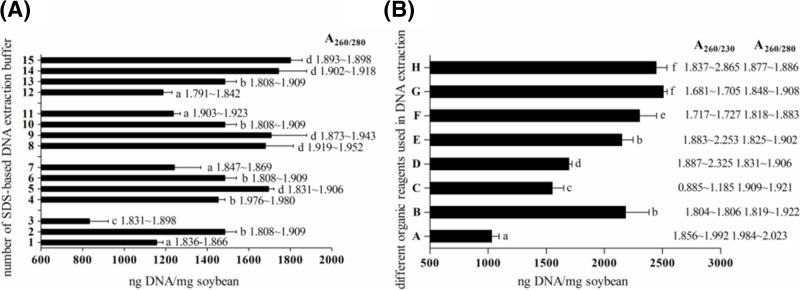
Optimization of the SDS-based method Effect of SDS lysis buffer components’ concentrations (**A**) and organic compounds (**B**) on DNA yield and purity during extraction from soybean seeds. Protocols of (A,B) correspond to SDS method 2 and SDS method 3 described in 2.2, respectively. 1, 2, 3, …, 14, and 15 in (A) and A, B, …, G, and H in (B) are detailed in Table 2; different lowercase letters indicate significant differences amongst DNA yields.

We investigated the effect of lysis buffer composition on DNA extraction efficiency. In general, surfactants such as CTAB and SDS are inclined to interact with polymers (protein, DNA etc.) driven by electrostatic, dipolar, and hydrophobic forces [20]. Interactions between SDS and cell membrane proteins will contribute to cell lysis promoting DNA extraction, while interaction with DNA inhibits subsequent amplification assays [21]. Therefore, a suitable SDS concentration is needed to guarantee high yield and purity of extracted DNA. The highest yield of extracted DNA was obtained with 2% (w/v) SDS (Figure 2A).

DNA yields were also affected by NaCl, Tris/HCl, and EDTA concentrations. A_260/280_ ratios of extracted DNA remained excellent regardless of NaCl, Tris/HCl, and EDTA concentrations (Figure 2A). DNA extraction buffers 5, 9, and 15 had the highest yields (Figure 2A), suggesting that 150, 50, and 100 mM are the most suitable concentrations for NaCl, Tris/HCl, and EDTA, respectively. It is worth noting that increasing NaCl concentration in SDS lysis buffer decreased DNA yield, while in CTAB lysis buffer it was reported that high NaCl promotes polysaccharide solubility during isopropanol extraction, resulting in higher quality DNA [22]. Additionally, NaCl (150 mM), Tris/HCl (10 and 50 mM), and EDTA (50 and 100 mM) in lysis buffer caused no significant differences in DNA yield (Figure 2A). Considering that 100 mM or more EDTA would lead to an excessively sticky mixture and large deviation in reagent preparation, it was decided to take 50 mM as the most suitable concentration of EDTA. In conclusion, the optimized composition of SDS lysis buffer (pH 8.0) consisted of 2% SDS (w/v), 150 mM NaCl, 50 mM Tris/HCl, and 50 mM EDTA for soybean seeds.

In addition, the SDS-based method also has potential for extracting DNA from highly processed soya matrix [16]. For instance, Edwards’ buffer (0.5% (w/v) SDS, 250 mM NaCl, 200 mM Tris/HCl, 25 mM EDTA, pH 8.0) proved to be more efficient for DNA extraction from highly processed soya matrix (soy sauce, soya milk etc.) than CTAB buffer [3]. It seems that SDS lysis buffers are particularly useful for separation of DNA from soya matrix, while the most suitable composition of lysis buffer depends on the processing degree of the soya materials.

### Optimization of organic reagents used in DNA extraction

Soybean contains approximately 40% protein (w/w) and 20% lipids (w/w), and any remainder lowers DNA purity and inhibits PCR amplification [6,23]. Accordingly, it was crucial to separate DNA from protein and lipids in the present study, instead of the polysaccharides, polyphenols, terpenoids, and tannins mainly found in mature fruit and plant tissues [24].

We studied the effect of organic compounds on separation of protein, oil, and polyphenols etc. from DNA. It has been demonstrated that phenol can dissolve protein dissociated from nucleic acids [25]. However, the specific gravity of purified phenol is only 1.07, similar to water, so would form a mixture with the aqueous phases or even invert when the phenol was used to extract protein. This limitation can be solved by mixing phenol with chloroform (1:1, v/v), a higher density reagent (1.47) [13]. Additionally, after dissolving the protein in phenol/chloroform (P: C, 1: 1, v/v), a protein-chloroform gel is formed, leaving nucleic acid in the aqueous supernatant for recovery [26]. Simultaneously, lipids dissolved into chloroform and isoamyl alcohol act as a foam-reducing agent. Accordingly, phenol, chloroform, and isoamyl alcohol were chosen as candidate reagents to purify DNA.

In the present study, protocols A (P: C: I) and B (C: I) were compared to evaluate their efficiency for DNA purification on the first extraction. Concentration of extracted DNA was higher in protocol B, indicating that C: I performed better at removing inhibitors from DNA solution (Figure 2B). According to the report by Green and Sambrook [13], P: C: I acted more efficiently than P:C for deproteinization. So, P: C extraction was not carried out in this study. Figure 2B also indicates that the absence of C: I in third extraction (protocol C) resulted in a much lower A_260/230_ ratio. This indicates lower practicability for PCR analysis [27]. So, C: I extraction was essential for purifying DNA after RNase was added.

Reagents used to precipitate DNA were also compared. We found that protocol D achieved significantly higher DNA yield than A, indicating the positive effect of metal cations, which neutralize the negatively charged sugar phosphate in the DNA backbone during DNA precipitation, as approved by Green and Sambrook [12]. Protocol E showed the best results for DNA isolation, proving that isopropanol was the most efficient compound for DNA first precipitation. Protocols B and F differed between ethanol and isopropanol use in the second precipitation step. Isopropanol gave a higher DNA yield, but a lower A_260/230_ ratio (Figure 2B). The positive effect on yield and negative effect on DNA purity were enhanced and verified by protocol G, with two isopropanol precipitations. Green and Sambrook [12] pointed out that the solubility of DNA in isopropanol is lower than in ethanol, accounting for the higher DNA yield with isopropanol, and that sucrose and mineral salts easily coprecipitate with DNA when isopropanol is used, generating lower quality extracted DNA. In general, ethanol is preferred for nucleic acid precipitation. According to the high performance of protocol H, it was concluded that isopropanol and ethanol for the first and second precipitation respectively were able to produce high yield and purity of extracted DNA.

In summary, the extraction and precipitation compounds used in raw soya DNA extraction were optimized to C: I, C: I, isopropanol, and ethanol corresponding to the first and third extraction, and first and second precipitation procedures, respectively.

### Suitability of the optimized DNA extraction method in different varieties of soybean seeds

**Figure 3:**
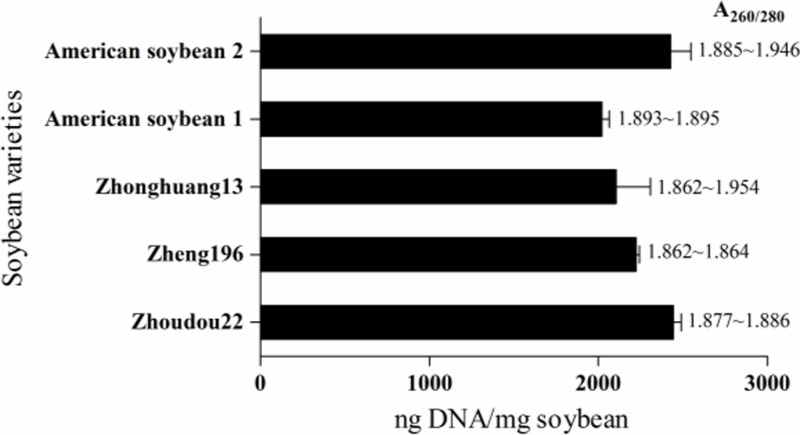
Verification of the optimized DNA extraction method

The optimized SDS-based DNA extraction method was applied to three varieties of non-transgenic soybean (Zhoudou 22, Zheng 196, and Zhonghuang 13) and two varieties of transgenic American soybean to evaluate its reliability and efficiency. DNA yields from the five soybean varieties fluctuated between 2020 and 2444 ng/mg soybean (Figure 3), a rather high yield compared with the approximately 50–350 ng/mg soybean obtained by other reporters [7,8,28] and equal to 1606–2300 ng/mg soybean [10]. Moreover, A_260/280_ ratios ranged from 1.862 to 1.954, revealing a clean DNA extract. These results indicate a wide suitability of the optimized DNA extraction method for raw soybean seeds.

### Sensitivity of the optimized DNA extraction method

**Figure 4:**
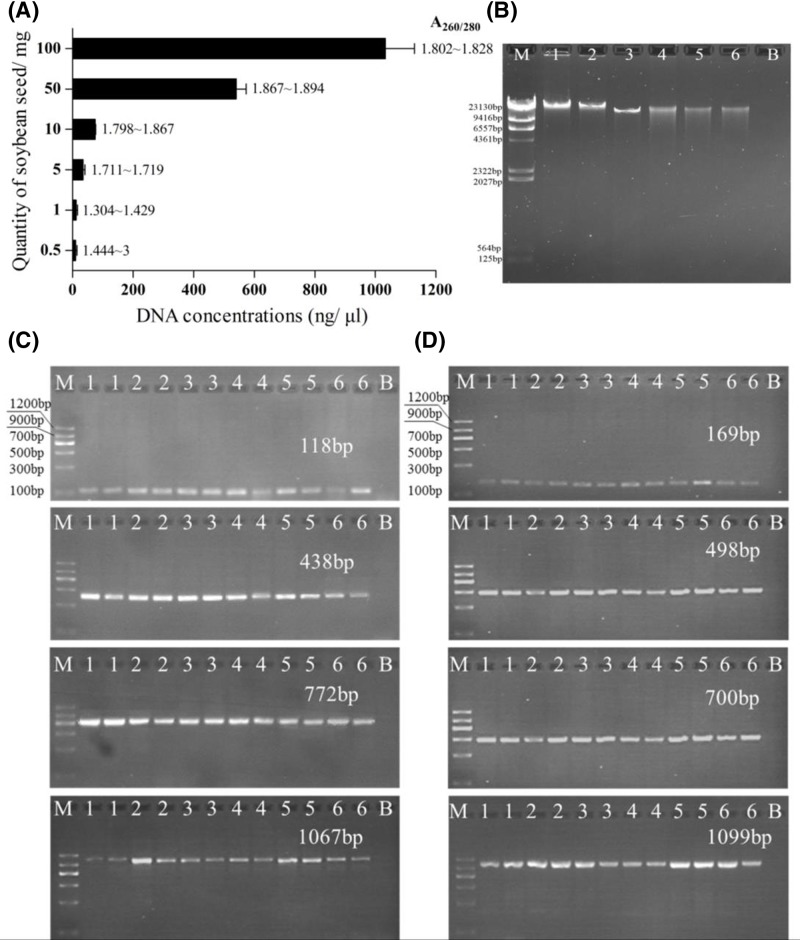
Performance of optimized DNA extraction method with RRS1 (**A**) DNA yield from different amounts of RRS1; (**B**), agarose gel electrophoresis of genomic DNA (gDNA) corresponding to (A); (**C**,**D**), agarose gel electrophoresis of different length PCR products amplified from the soybean *lectin* and *cp4epsps* genes respectively; M in (B), λ DNA HindIII Marker (TIANGEN, Beijing, China); M in (C,D), Marker II (TIANGEN, Beijing, China); lanes 1, 2, 3, 4, 5, and 6 represent gDNA or PCR products from 100, 50, 10, 5, 1, and 0.5 mg of RRS1 respectively; B, negative control of PCR reagents.

Different amounts of RRS1 were applied to DNA extractions to check the minimum quantity of soya material needed to obtain enough DNA for follow-up PCR assays. DNA yield increased in proportion to the amount of RRS1 (Figure 4A), which was consistent with the agarose gel electrophoresis results (Figure 4B). A DNA yield of 10.5 ng/μl was achieved even after the raw material quantity decreased to 0.5 mg. However, A_260/280_ ratios were below 1.8 when RRS1 quantity was 5 mg or lower, which indicates potential for unsatisfactory PCR amplification (Figure 4A). Accordingly, conventional PCR screening for the native *lectin* and *cp4epsps* genes was carried out to test the usability of extracted DNA, especially at low concentrations, for PCR amplification. Sequences ranging from approximately 100 to 1000 bp in length from endogenous and exogenous target genes were amplified in this work. Positive results were observed from all soybean samples both for the *lectin* and *cp4epsps* genes (Figure 4C,D), suggesting the practicability of extracted DNA from all ranges of RRS1 tested in the present paper. Thus, the minimum amount of RRS1 required for DNA extraction and successful PCR amplification was reduced to 0.5 mg by the optimized SDS-based method in this work.

## Conclusion

SDS-based DNA extraction was able to achieve the highest yield from raw soybean seed compared with the CTAB method and commercial kits (DP305 and DNeasy Plant Mini Kit). Then, optimization of the SDS-based method was performed step by step to maximize the DNA yield achieved from soybean seeds. The final SDS-based protocol included SDS lysis buffer (2% SDS (w/v), 150 mM NaCl, 50 mM Tris/HCl, 50 mM EDTA, pH 8.0), first and third extraction reagent (C: I), first precipitation reagent (isopropanol) and second precipitation reagent (ethanol). The suitability and high efficiency of the optimized SDS-based method were tested and verified by applying it to several other soybean varieties. In addition, sufficient amount of DNA was achieved from 0.5 mg soybean material using this method, enough to perform a successful PCR assay. The protocol obtained herewith will greatly facilitate DNA extraction from less processed soya matrix, especially for limited materials.

## Abbreviations

C: I: chloroform/isoamyl alcohol (24: 1, v/v)
EPSPS: 5-enolpyruvyl-shikimate-3-phosphate synthase
GM: genetically modified
P: C: phenol/chloroform (24: 1, v/v)
P: C: I: phenol/chloroform/isoamyl alcohol (25: 24: 1, v/v/v)
RRS: roundup ready soybean

## References

[1] Foreign Agricultural Service and United States Department of Agriculture (2018) Oilseeds: World Markets and Trade, PSD Data

[2] ISAAA (2017) Global Status of Commercialized Biotech/GM Crops in 2017: Biotech Crop Adoption Surges as Economic Benefits Accumulate in 22 Years. In ISAAA Brief No. 53, ISAAA, Ithaca, NY

[3] Turkec A., Lucas S.J. and Karlik E. (2016) Monitoring the prevalence of genetically modified (GM) soybean in Turkish food and feed products. Food Control 59, 766–772 10.1016/j.foodcont.2015.06.052

[4] The Commission of the European Communities (2003) Regulation (EC) No 1830/2003 of the European Parliament. Off. J. Eur. Union. L268, 24–28

[5] Costa J., Mafra I., Amaral J.S. and Oliveira M.B.P.P. (2010) Monitoring genetically modified soybean along the industrial soybean oil extraction and refining processes by polymerase chain reaction techniques. Food Res. Int. 43, 301–306 10.1016/j.foodres.2009.10.003

[6] Lo Y.-T. and Shaw P.-C. (2018) DNA-based techniques for authentication of processed food and food supplements. Food Chem. 240, 767–774 10.1016/j.foodchem.2017.08.022 28946341

[7] Mafra I., Silva S.A., Moreira E.J.M.O., Silva C.S.F.D., Beatriz M. and Oliveira P.P. (2008) Comparative study of DNA extraction methods for soybean derived food products. Food Control 19, 1183–1190 10.1016/j.foodcont.2008.01.004

[8] Demekea T., Holigroski M. and Phan A. (2012) Assessment of DNA extraction methods for PCR testing of discontinued or unapproved biotech events in single seeds of canola, flax and soybean. Food Control 24, 44–49 10.1016/j.foodcont.2011.09.001

[9] Stefanova P., Taseva M., Georgieva T., Gotcheva V. and Angelov A. (2013) A modified CTAB method for DNA extraction from soybean and meat products. Biotechnol. Biotec. Eq. 27, 3803–3810 10.5504/BBEQ.2013.0026

[10] Kamiya M. and Kiguchi T. (2003) Rapid DNA extraction method from soybean seeds. Breed Sci. 53, 277–279 10.1270/jsbbs.53.277

[11] Xu W.-T., Huang K.-L., Deng A.-K., Liang Z.-h. and Luo Y.-B. (2007) Variations of tissue DNA density and nuclear DNA content in soybean lines and their impacts on the GMO quantification. Food Control 18, 1300–1306 10.1016/j.foodcont.2006.08.009

[12] Green M.R. and Sambrook J. (2016) Precipitation of DNA with Ethanol - A Laboratory Manual, Cold Spring Harbor Laboratory Press, U.S.A. 10.1101/pdb.prot093377 27934690

[13] Green M.R. and Sambrook J. (2017) Isolation of High-Molecular-Weight DNA Using Organic Solvents - A Laboratory Manual, Cold Spring Harbor Laboratory Press, U.S.A. 10.1101/pdb.prot093450 28373491

[14] Dellaporta S.L., Wood J. and Hicks J.B. (1983) A plant DNA minipreparation: version II. Plant Mol. Biol. Rep. 1, 19–21 10.1007/BF02712670

[15] International Standard (ISO) 21571 (2005) Foodstuffs-Methods of analysis for the detection of genetically modified organisms and derived products-nucleic acid extraction, International Organization for Standardization, Switzerland, CH

[16] Wang X., Teng D., Tian F., Guan Q. and Wang J. (2012) Comparison of three DNA extraction methods for feed products and four amplification methods for the 5′-junction fragment of Roundup Ready soybean. J. Agric. Food Chem. 60, 4586–4595 10.1021/jf300827q 22515503

[17] Tian F., Guan Q., Wang X., Teng D. and Wang J. (2014) Influence of different processing treatments on the detectability of nucleic acid and protein targets in transgenic soybean meal. Appl. Biochem. Biotech. 172, 3686–3700 10.1007/s12010-014-0760-2 24566925

[18] Elsanhoty R.M., Ramadan M.F. and Jany K.D. (2011) DNA extraction methods for detecting genetically modified foods: a comparative study. Food Chem. 126, 1883–1889 10.1016/j.foodchem.2010.12.013 25213972

[19] Milia M., Vodret B., Serratrice G., Soro B. and Mancuso M.R. (2008) Three different extraction methods for detecting roundup ready soybean in processed food from the Italian market. Int. J. Integr. Biol. 3, 123–130

[20] Chatterjee A., Moulik S.P., Majhi P.R. and Sanyal S.K. (2002) Studies on surfactant-biopolymer interaction. I. Microcalorimetric investigation on the interaction of cetyltrimethylammonium bromide (CTAB) and sodium dodecylsulfate (SDS) with gelatin (Gn), lysozyme (Lz) and deoxyribonucleic acid (DNA). Biophys. Chem. 98, 313–327 10.1016/S0301-4622(02)00107-2 12128183

[21] Perumal N.V., Zhang X., Yuki M., Fumio I. and Wang F. (2016) A modified SDS-based DNA extraction method for high quality environmental DNA from seafloor environments. Front. Microbiol. 7, 1–13 27446026 10.3389/fmicb.2016.00986 PMC4917542

[22] Sahu S.K., Thangaraj M. and Kathiresan K. (2012) DNA extraction protocol for plants with high levels of secondary metabolites and polysaccharides without using liquid nitrogen and phenol. Int. Math Scho. Notices 2012, 1–6 10.5402/2012/205049 PMC4890884 27335662

[23] Harrigan G.G., Ridley W.P., Riordan S.G., Nemeth M.A., Sorbet R., Trujillo W.A. (2007) Chemical composition of glyphosate-tolerant soybean 40-3-2 grown in europe remains equivalent with that of conventional soybean (Glycine max L. J. Agric. Food Chem. 55, 6160–6168 10.1021/jf0704920 17608426

[24] Porebski S., Bailey L.G. and Baum B.R. (1997) Modification of a CTAB DNA extraction protocol for plants containing high polysaccharide and polyphenol components. Plant Mol. Biol. Rep. 15, 8–15 10.1007/BF02772108

[25] Kirby K.S. (1956) A new method for the isolation of ribonucleic acids from mammalian tissues. Biochem. J. 64, 405–408 10.1042/bj0640405 13373784 PMC1199750

[26] Sevag M.G., Lackman D.B. and Smolens J. (1938) The isolation of the components of streptoeoeeal nueleoproteins in serologieally active form. J. Biol. Chem. 124, 42–49

[27] Matsuoka T., Kuribara H., Akiyama H., Miura H., Goda Y., Kusakabe Y. (2001) A multiplex PCR method of detecting recombinant DNAs from five lines of genetically modified maize. J. Food Hyg. Soc. Jpn. 42, 24–32 10.3358/shokueishi.42.24 11383153

[28] Agbagwa I.O., Datta S., Patil P.G., Singh P. and Nadarajan N. (2012) A protocol for high-quality genomic DNA extraction from legumes. Genet. Mol. Res. 11, 4632–4639 10.4238/2012.September.14.1 23079974

